# LncRNAs in breast cancer: a link to future approaches

**DOI:** 10.1038/s41417-022-00487-w

**Published:** 2022-07-04

**Authors:** Nikolaos Sideris, Paola Dama, Salih Bayraktar, Thomas Stiff, Leandro Castellano

**Affiliations:** 1 Department of Biochemistry and Biomedicine, School of Life Sciences, University of Sussex, Falmer, Brighton, BN1 9QG UK; 2 Division of Cancer, Department of Surgery and Cancer, Imperial College London, London, SW7 2AZ UK

**Keywords:** Breast cancer, Molecular biology

## Abstract

Breast cancer affects millions of women each year. Despite recent advances in targeted treatments breast cancer remains a significant threat to women’s health. In recent years the development of high-throughput sequencing technologies has advanced the field of transcriptomics shedding light on the role of non-coding RNAs (ncRNAs), including long ncRNAs (lncRNAs), in human cellular function and disease. LncRNAs are classified as transcripts longer than 200nt with no coding potential. These transcripts constitute a diverse group of regulatory molecules essential to the modulation of crucial cellular processes, which dysregulation of leads to disease. LncRNAs exert their regulatory functions through their sequences and by forming complex secondary and tertiary structures that interact with other transcripts, chromatin and/or proteins. Numerous studies have provided evidence of the involvement of LncRNAs in tumor development and disease progression. They possess multiple characteristics that make them novel therapeutic and diagnostic targets. Indeed, the discovery of a novel mechanism by which lncRNAs associated with proteins can induce the formation of phase-separated droplets broadens our understanding of the spatiotemporal control of cellular processes and opens up developing a new treatment. Nevertheless, the role and the molecular mechanisms of many lncRNAs in the regulation of cellular processes and cancer still remain elusive. This is due to the absence of a thorough characterization of the regulatory role of their loci and the functional impact of their aberrations in cancer biology. Here, we present some of the latest advances concerning the role of LncRNAs in breast cancer.

## Introduction

Data from the World Health Organization (WHO) indicates breast cancer as the most frequent malignancy affecting women worldwide [1]. It is a leading cause of mortality in developing countries and the second leading cause of cancer death in American women. In 2020 alone, the WHO recorded over 2.3 million breast cancer cases among women globally and 685,000 deaths, even if there is a significant variation in estimated incidence rates worldwide with a remarkable difference between Australia, North America, Europe and the rest of the world. The difference in mortality rates however are less pronounced [2]. Breast cancer is a very dynamic disease that occurs due to genetic and environmental queues.

Breast cancer exhibits great heterogeneity at both molecular and clinical levels. Each subtype presents varying biological traits with distinct pathological features and different clinical outcomes impacting the treatment planning [3]. Genetic/epigenetic data and protein markers are general criteria to classify breast tumors into subtypes [4].

Breast tumors are historically classified based on specific molecular signatures such as Progesterone Receptor (PR), Estrogen Receptor (ER) and Human Epidermal Growth Factor Receptor 2 (HER2) by classical immunohistochemical assay [5]. Further classification has been accomplished with the use of transcriptional profiling methods of a large set of tumors, revealing five major molecular subtypes i.e. luminal A, luminal B, HER2 over-expression, basal and normal-like tumors. Breast malignancies usually start out from luminal or basal cells of the duct lobular units of the breast. Tumor subtypes represent different biological entities consistent with the cell type of origin where the gene expression profile mirrors the molecular complexity of the tumors [6, 7]. Based on specific molecular subtypes the stratification of the patients results in distinct clinical outcomes and responses to the treatment [6, 7]. Of those, Luminal tumors (A and B) are primarily ER-positive with a slower rate of expansion but a larger incidence of relapse [8]. These are treated with a combination of chemotherapy and endocrine therapy to counteract the hormone receptor overexpression [9]. The HER2 positive subtype is marked by overexpression of the HER2 gene and poor prognosis. Treatment involves chemotherapeutic agents and targeted approaches aimed specifically against HER2 [10]. The basal-like subtype most commonly observed in triple-negative tumors, is the most aggressive subtype with the worst patient outcome [11]. When diagnosed at the earlier stages it can be cured in approximately 80% of patients [12–14].

Unfortunately, a significant percentage of women diagnosed with early-stage breast cancer experience the development of more aggressive phenotype months or even years after the initial treatment. One of the causes of refractory cancer is the cellular heterogeneity of the tumor. For example, cancer stem cells are subpopulation of cells within the tumor that do not respond to the conventional treatment, even the current approaches fall short when it comes to the more aggressive subtypes [15]. Furthermore, distant organ metastasis is the greatest challenge to modern oncotherapy, with invasive breast cancer being characterized as incurable despite current treatments, making the development of new more efficient therapies essential [16–18].

The development of next generation sequencing technologies enabled the accurate characterization of the human genome. Despite the fact that the majority of nucleotides are transcribed under specific conditions, it has been proven that only a mere fraction of the transcribed genome encodes for proteins. Indeed, the associated transcriptome—which is found pervasively transcribed, led to the discovery of a new class of non-coding transcripts, named long non-coding RNAs (lncRNAs) [19], indicating that the majority of transcripts may function as non-coding molecules [20].

This new subset of RNA molecules has been found to act primarily as gene expression regulators [19]. There is mounting evidence that dysregulation of lncRNA loci can devastate the normal transcriptional landscape resulting in aberrant gene expression and ultimately malignant transformation. A thorough characterization of this new species of RNA could provide insights into new avenues of therapy and disease management. The fundamental characteristics of these molecules such as their highly specific expression patterns and functional tertiary structure make them ideal for use as diagnostic biomarkers, and promising targets for the development of pharmacological inhibitors. In this review we will survey the latest breakthroughs regarding the involvement of lncRNAs in breast cancer and explore their clinical potential.

## Non-coding RNAs (ncRNAs)

NcRNAs have been classified into two groups based on the size of the transcripts. LncRNAs are transcripts longer than 200 nucleotides [21]. Similar to mRNAs, lncRNAs are directly transcribed from genes and can undergo alternative splicing to produce numerous isoforms [22, 23]. Further similarities between lncRNAs and mRNAs on the transcript level include transcription by RNA-polII, a 5’ end cap and a 3’ poly-A tail, loci exhibiting all the aspects of a bona fide protein coding gene including promoter conservation, indicative chromatin structure and regulation via transcription factors and epigenetic remodelers [24, 25].

However, lncRNAs possess some fundamental differences which make them unique. When compared to protein-coding genes, apart from their lack of protein coding potential, lncRNAs exhibit relatively lower levels of expression and poor evolutionary conservation among species [19, 20, 25]. The most distinctive characteristic they possess however, would be their ability to exert their regulatory functions through elaborate structures [26–28]. Interestingly, while conservation of their primary sequence varies and is generally poor, many lncRNAs present conservation at the tertiary structure level [29, 30]. It is also worth noting that lncRNAs are expressed in most tissues and cell types (including but not limited to stem cells, immune cells, brain cells, tumor cells). Generally, the overall tissue expression levels of lncRNAs are lower compared to those of mRNAs. Nevertheless, lncRNAs can be highly expressed, and are easy to detect in some cell types; demonstrating higher cell/tissue specific patterns of expression than those of protein coding genes, a finding consistent with their role in regulating the cell’s transcriptional landscape [31, 32]. These transcripts are involved in regulating post-transcriptional activity, chromatin remodeling, mRNA integrity and protein interactions amongst others. Therefore, ultimately being responsible for coordinating essential processes like: metabolism, development and differentiation [33–35].

## Localization of LncRNAs

LncRNAs have been shown to localize both in the nucleus and cytoplasm, acting through a wide range of mechanisms with distinct but equally important functions. However, the underlying mechanisms of most lncRNAs remains a mystery.

A significant number of lncRNAs function exclusively in the nucleus. Their expression dysregulation can wreak havoc on cellular homeostasis and lead to malignant transformation.

**Fig. 1:**
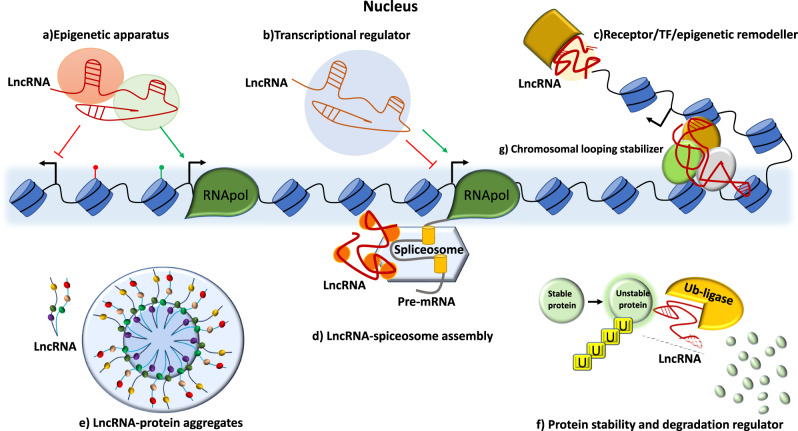
Schematic overview of LncRNA mechanisms in the nucleus. **a** Recruitment of epigenetic apparatus members for expression regulation of the epigenetic level (HOTAIR, PANDAR etc). **b** Interaction with transcription machinery for regulation on the transcriptional level. **c** Interacting with proteins to enhance signaling (lnc-BM) or acting as molecular decoys to inhibit DNA-protein interactions (Meg3). **d** Assembly of spliceocome for nascent transcript modification regulation (MALAT1). **e** Interaction with proteins to form RNA-protein aggregates and assemble membraneless organelles through llps such as paraspeckles (NEAT1). **f** Regulating protein stability and degradation (ANCR). **g** Facilitate and stabilize chromosomal looping to bridge distal enhancer elements with gene promoters.

Notable mechanisms in the nucleus involve interactions with epigenetic remodelers, transcription factors and spliceosomes, where the lncRNAs act as guides, scaffolds or stabilizers to influence chromatin architecture alteration and gene expression [36, 37] (Fig. 1).

More specifically, lncRNAs in the nucleus can guide transcription factors and epigenetic remodelers to their target genes, or even sequester them to promote or inhibit target gene expression, while also controlling protein activities in the nucleus by regulating their ubiquitination and mRNA stability. LncRNAs are also capable of altering chromatin in 3D-space by initiating and maintaining chromosomal looping to bridge distant enhancer elements and gene promoters (Fig. 1).

Some of the best- documented examples include HOTAIR and MALAT1. HOTAIR regulates the expression of the HOX gene cluster by guiding PRC2 and GAS5. These interact directly with activated glucocorticoid receptors, thus preventing the binding of the target genes to them [37], blocking the receptors from binding their target genes and MALAT1, which regulates alternative splicing via controlling serine/arginine splicing factor phosphorylation [38–40].

lncRNAs in the cytoplasm regulate mRNA stability by directly controlling de-adenylation, as well as protecting mRNA transcripts from miRNA mediated degradation by acting as molecular decoys, a process known as miRNA sponging [41, 42]. A novel example of this mechanism is lncRNA SNHG7 which promotes tumourigenesis by acting as a competing endogenous RNA, sequestering a number of tumor suppressor miRNAs in a variety of cancers [43, 44].

## lncRNA and epigenetics crosstalk

The earliest and perhaps best described example of nuclear lncRNA would be Xist, which facilitates X-chromosome inactivation by interacting with and guiding methyltransferases to the X chromosome in females [45]. Interestingly a new study has also shown that Xist can regulate cell proliferation and migration in breast and ovarian cancer by mediating macrophage polarization through competition with miR-101 for the regulation of C/EBPα and KLF6 [46]. HOTAIR, as mentioned earlier, is another well characterized nuclear lncRNA which binds to the PRC2 complex of the epigenetic apparatus to modulate histone modifications of target genes in the HoxD cluster in trans, thus conferring transcriptional silencing [47]. It has been shown that HOTAIR is capable of altering the state of chromatin in tumor metastasis, and has been found to be upregulated in metastatic breast carcinomas resulting in an altered pattern of PRC2 occupancy from breast epithelial cells to that of embryonic fibroblasts [48, 49].

Regulation of the transcriptional landscape facilitated by interactions between lncRNAs and chromatin remodelers such as members of the polycomb complex are a common function of nuclear lncRNAs in cancer [50]. A recent study has shown that overexpression of lncRNA PANDAR in breast cancer cells promotes cell proliferation by regulating G1 to S phase transition. Knockdown of the lncRNA induced cell cycle arrest at the G1 phase. Further chromatin and RNA immunoprecipitation (ChiP and RiP) experiments showed PANDAR to interact with the Bmi1 component of the PRC1 complex to downregulate the expression of p16^IK4A^, a known cell cycle regulator, by facilitating Bmi1 binding to the p16 promoter [51, 52]. Similarly, researchers have discovered an overexpressed oncogenic lncRNA in ER-negative breast cancer: linc00511. linc00511 worsens patient prognosis by inhibiting apoptosis and accelerating the G1/S transition. This effect is achieved by repressing CDKN1B expression; the gene which encodes for the p27 tumor suppressor protein [53]. Overexpression of linc00511 is shown to be triggered directly by the deficiency of ER and activated by the TFAP-2 transcription factor. EZH2, the catalytic subunit of PRC2, is recruited by linc00511 to the promoter of CDKN1B. Silencing of the lncRNA suppressed tumor growth in mice while in vitro CHiP assays confirmed that the knockdown inhibited EZH2 association with the CDKN1B promoter and limited the deposition of H3K27me3 without affecting EZH2 expression [54].

The flexibility of lncRNA mediated regulation and their functional variability in tumourigenesis has been observed through studying the upregulation of lncRNA TINCR by STAT3 [55]. Through bioinformatic analyses utilizing the GEPIA tool, researchers observed TINCR to be significantly upregulated in various cancer types. TINCR correlated to poor prognosis in breast cancer patients with EGFR involvement. A series of in vitro and in vivo knockdown experiments showed that TINCR promoted tumourigenesis via upregulation of EGFR. TINCR was found to be present and active in both the cytoplasm and the nucleus. In the cytoplasm TNCR acted as a ceRNA (competing endogenous RNA) to sponge miR-503-5p, which downregulates both EGFR mRNA and TINCR. In the nucleus however, bisulfate sequencing revealed that TINCR epigenetically silenced the miRNA by recruiting DNMT1 to its promoter and thus creating a positive feedback loop for the expression of EGFR and the lncRNA itself [55].

**Fig. 2:**
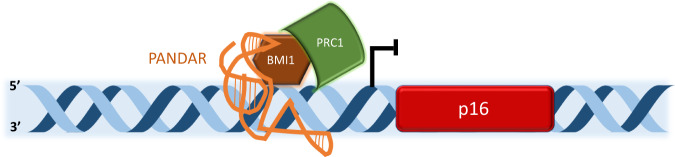
Representative example of lncRNA and epigenetics crosstalk. PANDAR interacts with BMI1 to recruit PRC1 to the p16 promoter leading to downregulation of gene expression.

Thorough investigation into the impact of the lncRNA Stem Cell Inhibitory Transcript (SCIRT) on tumor-initiating cell (TIC) transcriptional programs in 3D breast cancer cultures yielded some rather intriguing data, demonstrating the complexity of lncRNA regulatory circuits [56–58]. TICs are slow proliferating and therefore widely resistant to chemotherapy. TICs are also highly metastatic cells with stemness properties capable of promoting tumor heterogeneity [59–61]. RNA-seq of cells cultured in adherent and sphere conditions at specific timepoints showed a reduction of proliferative gene expression, accompanied by an increase in self renewal expression signatures during the transition to spheres. The NGS data suggested SCIRT as a possible regulator of this process. Knockdown and overexpression assays revealed SCIRT’s role as a tumor suppressor; it can restrain stemness in vitro, and tumor formation in mice. Combined with analysis of ChiP data, a series of Capture Hybridization Analysis of RNA Targets (CHART)-seq, RIP, epigenetic mark screening by ChiP-seq experiments, revealed that SCIRT binds globally to promoter/enhancer regions to increase cell-cycle gene expression and decrease self-renewal gene expression by interacting with EZH2 (catalytic component of PRC2), SOX2 (TF essential for self-renewal) and FOXM1 (TF critical for proliferation associated transcription). On the molecular level, SCIRT utilizes a G4-quadruplex in its 5’ region to bind EZH2 and colocalizes with EZH2 and SOX2 at CpG islands of target gene promoters. Mechanistically SCIRT directly antagonizes EZH2 and SOX2 activity at the promoters of stemness related genes tipping the scale towards self-renewal suppression. On the other hand, SCIRT increases the affinity of EZH2 for FOXM1 at the promoters of cell-cycle promoting genes, thus recruiting FOXM1 in a protein-protein interaction dependent manner to overcome EZH2-mediated expression repression. SCIRT appears to be overexpressed in aggressive breast cancer samples according to TCGA and GTEX cohort datasets, conferring a more favorable outcome [56]. Taken together, these findings provide a novel regulatory network that could be further utilized with great implications in a clinical setting, and lead to new prognostic and therapeutic targets (Fig. 2).

## lncRNA interactions with TF and transcription mediators

Apart from their significant interaction with chromatin remodelers, lncRNAs are also capable of associating with transcription factors and mediators to exert their oncogenic functions with great implications to disease progression. A novel mechanism of breast cancer brain metastasis promotion was discovered, where lnc-BM seems to play a critical role in JAK/STAT signaling in a mutation independent manner. Jak2 is a non-receptor tyrosine kinase involved in cell growth and proliferation control, frequently mutated in cancers [62]. In many cases Jak2 hyperactivation leads to the promotion of oncogenic inflammation pathways through the phosphorylation of STAT3 [63]. Researchers have found that lnc-BM plays an important part in breast cancer brain metastasis, via coordinating cell to cell communication between breast cancer cells and the brain microenvironment. lnc-BM was observed to directly bind to JAK2, conferring a more active structural conformation to the kinase, this coincides with enhanced JAK2/STAT3 signaling in BCBM. This hyperactivated signaling leads to upregulation of ICAM1 promoting cancer cell adherence in the brain, and the secretion of CCL2 which attracts macrophages to the lesion, prompting them to secrete signal enhancing mediators [64]. Remarkably while lnc-BM could promote metastasis in murine models, nanoparticle encapsulated siRNAs were successful in treating the disease via lnc-BM downregulation, thus providing a potential new therapeutic approach. Another notable example of lncRNA dependent phosphorylation was observed in in vitro and in vivo TNBC experiments where DANCR was shown to bind RXRA and increase its association with glycogen synthase kinase-3b (GSK-3b), thus increasing serine phosphorylation of RXRA and promoting tumourigenesis via enhanced PI3K/AKT signaling [65].

One of the main characteristics of lncRNAs is a general lack of conservation at the primary sequence level among species, although there are some exceptions [30–66]. Researchers have identified the nuclear enriched Linc01271 as the human ortholog of murine MATAR25, and linked it to metastatic invasion in breast cancer with poor patient prognosis through regulation of TNS1 expression. TNS1 is strongly suppressed in cancer, especially metastatic cancer, and is a key component of fibrillar adhesions, and positively modulates cell migration and invasion [67, 68]. MATAR25 knockouts in aggressive breast cancer cell lines generated via CRISPR-Cas9 lead to a reduction in cell proliferation, migration, and invasion capabilities. These findings were corroborated by decreased tumor progression and metastasis in tumor bearing KO-mice. Conversely, restoring the expression of MATAR25 restored the proliferative and invasive phenotype in mice.

A combination of MATAR25-KO cell RNA-seq and CHIRP-seq data found TNS1 to be the most promising downstream target, further confirmed by its expression levels during these conditions [69]. On a molecular level, MATAR25 exerted its function by binding to PURB, a transcriptional co-activator, by acting as a scaffold for the lncRNA/PURB/TNS1 interaction, as confirmed by antisense oligonucleotide pulldown (RAP) and PURB-KO experiments. Genome synteny studies identified Linc01271 as a potential ortholog in humans, which was then confirmed when ectopic expression of Linc01271 in mouse MATAR25-KO cells rescued the proliferative and invasive phenotype. The high level of expression of Linc01271 in metastatic breast cancer according to TCGA, in combination with successful tumor size reduction in MATAR25-ASO mediated silencing in mice, highlights Linc01271 as a prominent therapeutic target requiring further investigation [69].

**Fig. 3:**
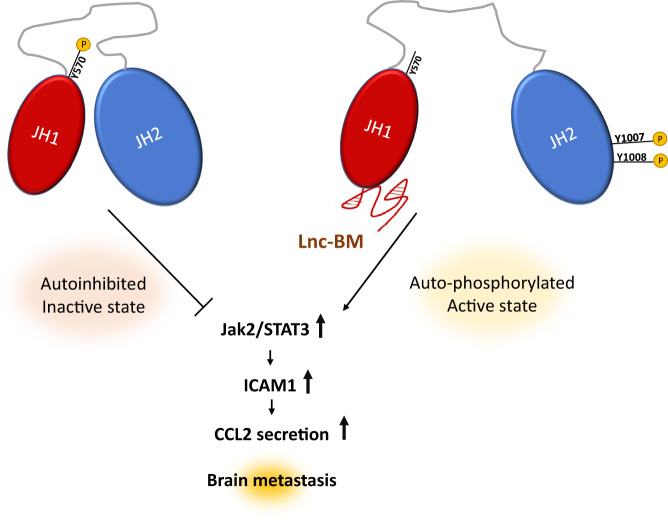
Visual representation of lncRNA-protein interactions. Lnc-BM directly binds to JAK2 conferring a more active state and enhancing signaling activity.

In another study nuclear lncRNA EGOT1 was found to be downregulated in breast cancer tissues and its involvement in in microtubule-associated function hinted to a potential impact on paclitaxel treatment, a microtubule disruptor utilized as a chemotherapeutic agent [70]. Overexpression of EGOT1 in mouse xenografts was shown to sensitize the cells to paclitaxel, resulting in reduced tumor volume and weight, while its downregulation seemed to protect the cells. EGOT1 is transcribed from the intronic regions of the ITPR1 gene in an antisense direction, its expression was positively correlated with ITPR1 mRNA levels. Further investigation showed overexpression of EGOT1 induced autophagy through increased IPTR mediated autophagic signals and vesicles, while EGOT1 knockdown had the opposite effect. Ectopic expression of EGOT1 upregulated endogenous pre-ITPR1 mRNA, and increased stability of the pre-mRNA. RNA-FISH and treatment with RNAse-H subsequently revealed that EGOT1 could directly hybridize with the IPTR1 pre-mRNA, forming a dsRNA to regulate ITPR1 expression. Interestingly, RIP results showed that the dsRNA could physically associate with the RNA binding protein hnRNP1 through a specific region of EGOT1. Knockdown of hnRNP1 reduced EGOT1 and ITPR1 transcript levels, potentially implicating it in regulation of alternative splicing. This data demonstrated the ability of EGOT1 to function both in cis and in trans for the regulation of ITPR1 expression thereby sensitizing breast cancer cells to paclitaxel [70] (Fig. 3).

## lncRNA as chromatin architects

Many previous studies have focused on elucidating the functional impact of lncRNA-protein interactions both in the nucleus and the cytoplasm. Yet, their ability to associate with double stranded DNA has remained relatively unexplored [68]. lncRNAs have been observed to interact with and bind to specific double stranded DNA regions. Through poly-purine sequence-specific recognition and hydrogen bond interactions, they form RNA-DNA triplex structures thanks to specific triplex-forming oligonucleotide sequences (TFOs) [71–73]. These triplex formation interactions have been reported to play a role in the regulation of gene expression in cancer, with the potential of providing new targets for therapeutic approaches [73, 74].

In 2019 a comprehensive investigation into the ability of lncRNA MIR100HG to promote TNBC cell proliferation revealed its capability of forming an RNA-DNA triplex at the promoter of the p27 gene to regulate its expression [75]. Overexpression and knockdown of the lncRNA in vitro showed increased cell proliferation and cell cycle arrest at the G1 phase, with similar results occurring in mouse xenografts. RNA-seq revealed p27 to be among the genes affected by MIR100HG silencing, resulting in reduction at both the transcript and protein level. Predictive bioinformatic analysis provided three TFOs (namely TFO1-3) present in the lncRNA sequence, and a triplex targeting site in the 5’UTR of the p27 gene. On a molecular level it was revealed that p27 regulation by MIR100HG was TFO1 dependent. TFO1 binding and triplex forming ability was observed in vitro and in vivo via chromatin isolation by RNA purification (CHIRP) of biotin labeled TFO1 in TNBC cell lysates. This showed significant enrichment of p27 and TFO1, providing a novel mechanism by which MIR100HG could potentially recruit chromatin remodelers or TFs to the promoter of p27 [75]. An earlier example of this mechanism was observed during a thorough examination of the function of MEG3 in breast cancer. It was revealed that MEG3 regulated the TGF-β pathway, where it exerts its function through the formation of an RNA-DNA triplex at the TGFR1 promoter via a GA-rich motif, facilitating the formation of an R-loop between the promoter and distal regulatory elements [76].

Apart from the functional role of lncRNA transcripts in gene expression regulation, it appears that lncRNA regulatory elements have an independent role of their own. Investigation of the role of the lncRNA PVT1 gene has yielded some rather intriguing results. PVT1 has been implicated in the promotion of tumourigenesis and metastasis in various cancers. In gallbladder cancer PVT1, recruiting DNMT1 and EZH2, promoted the methylation of miR-18b-5p leading to epigenetic silencing [77]. In Clear Cell Renal Cell Carcinoma (ccRCC) a PVT1/HIF2a positive feedback loop has been demonstrated, whereby PVT1 stabilizes HIF2a bound to the enhancer to transactivate its expression [78].

In triple negative breast cancer, PVT1 interacts with KLF5 and enhances its binding to the BAP1 de-ubiquitinase, increasing its stability, and promoting TNBC cell growth through beta catenin signaling upregulation [79]. However, what is truly remarkable about this gene is the recent discovery of its promoter’s capability to function as an autonomous regulator of c-MYC expression, independently from the lncRNA itself.

**Fig. 4:**
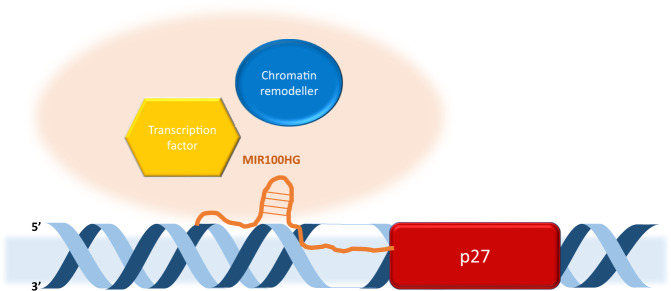
Visual representation of lncRNA influencing chromatin structure via RNA-DNA interactions. Example MIR100HG. Regulates p27 expression by forming a DNA-RNA triplex structure with the 5’ UTR of the gene possibly to recruit transcription regulators and chromatin modifiers to the site.

In sharp contrast to the oncogenic activity the PVT1 transcript demonstrates, a combination of 4C-seq, ATAC-seq and Hi-CHiP experiments has proven that promoter of PVT1 can act independently in breast cancer as a tumor suppressor by limiting c-MYC activity as it competes with the c-MYC promoter for a group of enhancers [80, 81]. Moreover, the extremely high frequency of PVT1 promoter mutations in numerous cancer types indicates its significance to the regulatory process [82]. In conclusion, this novel example shows that lncRNA mediated regulation is not limited to the transcript level. Regulatory elements of lncRNAs can act independently, have their own significant role to play in transcriptional regulation, and have provided a new insight into transcriptional regulatory processes (Fig. 4).

## LncRNAs as mediators of phase separation

Extensive studies have provided a new framework for how biological matter can be organized within the cell, through formation of biomolecular condensates enriched in RNA and proteins, which then form a number of membraneless organelles (cajal bodies, paraspeckles, RNP granules among others) to mete out a series of biological functions [83, 84]. Known as liquid-liquid phase separation (LLPS) or simply as phase separation, this phenomenon results from the interaction of proteins containing peptide segments with insufficient hydrophobic amino acids to mediate co-operative folding. Aforementioned proteins can react with each other or repetitive nucleic acid sequences, forming aggregates with defined boundaries [84, 85]. These droplet-like compartments allow for spatiotemporal control of various biochemical reactions and cellular functions, including signal transduction, RNA splicing and chromatin organization [86–88]. For example, it has been observed that the TAZ transcription factor and other TFs can facilitate gene expression by compartmentalizing the transcription machinery through LLPS activity of specific domains [89, 90]. Despite the early stages of LLPS research, accumulating evidence suggests its importance in cellular homeostasis and the impact of aberrant condensates in human pathologies. These include: neurodegeneration, cancer, and infectious diseases, where lncRNAs are emerging as potent modulators [91–93].

A well-established example of lncRNA mediated phase separation occurs via NEAT1, a transcript pivotal for the assembly and maintenance of paraspeckles in the nucleus, whose aberrations have been described in various cancers including breast [94–96]. A recent study revealed that NEAT1 possesses redundant sequences in its middle domain, these are used to recruit NONO protein dimers to initiate the oligomerization of DBHS proteins for the formation of an RNP complex, which during phase separation assembles the paraspeckle. Functional analysis of these subdomains showed they were essential to the assembly process, as deletion mutants were incapable of recruiting NONO, while assessment of aggregate formation exhibited their ability to induce a higher order assembly of paraspeckle proteins [97]. lncRNA SNHG9, a transcript which can interact and bind to phosphatidic acid (PA), has been clinically correlated with disease progression and poor prognosis in breast cancer with enhanced YAP activity, by decreasing phosphorylation through LATS1, a member of the Hippo pathway. SNHG9 was found to control the kinase activity by interacting with PA and binding to LATS1 through the C-terminal to facilitate protein aggregation, thus promoting phase separation via the formation of liquid droplets in a dose dependent manner [98].

Interrogation of the role of the interaction between lncRNA DIGIT and BRD3 has yielded some interesting findings with regards to transcriptional regulation in endoderm differentiation. CHiP-seq screening showed BRD3 recognizes and binds to H3K18ac throughout the genome and facilitates gene expression by occupying enhancer elements, while its capacity to form phase-separated droplets was demonstrated with titration in vitro. Researchers discovered that DIGIT interacts with BRD3 through its bromodomains, guiding it to specific genes involved in driving endoderm differentiation, where it promotes the formation of BRD3 aggregates to modulate transcriptional activity. Loss of BRD3 blocked differentiation, and similarly depletion of DIGIT blocked differentiation by impairing BRD3 to key target genes [99]. In a different study damage-induced lncRNAs (dilncRNAs) transcribed at sites of double stranded breaks, were found to recruit DNA-damage response (DDR) proteins like 53BP1 and assemble LLPS condensates in the form of DDR foci for the regulation of DSB signaling. ASO-mediated knockdown of the dilncRNAs attenuated the formation of the DDR foci and blocked DSB repair, highlighting their crucial role in modulating this process (Table 1) [100].

**Table 1:** List of biological relevant lncRNAs in Breast Cancer and their mechanisms-functions.

lncRNA	Mechanism	Localization	Expression Rate	Effectors	Function/Pathways	Biological Processes	Reference
HOTAIR	Epigenetic Crosstalk	Nucleus	Increased	HOX Gene cluster: PRC2 and GAS5	Chromatin remodeling	EMT transition and Metastasis	[38, 40]
MALAT1		Nucleus	Increased	SRSF1, TDP43, and PRC2 components, including EZH2	Alternative splicing; serine/arginine phosphorylation	Metastasis and tumor progression	[39, 132]
Xist		Nucleus	Decreased	C/EBPα and KLF6	Macrophage polarization	Cell proliferation and migration	[45, 46]
PANDAR		Nucleus	Increased	BMI1/P16^Ik4a^/C/EBPα/KLF6	Regulation of G1 to S phase transition	Cell proliferation and Cell cyle	[51, 52]
linc00511		Nucleus	Increased	CDKN1B	G1/S transition	Apoptosis inhibition	[53]
TINCR		Cytoplasm/Nucleus	Increased	EGFR/miR-503-5p	ceRNA/DNMT1 recruitment	Tumorigenesis	[55]
SCIRT		Nucleus	Increased	EZH2; SOX2; FOXM1	Self renewal expression signatures	Cell Cycle	[56–58]
lnc-BM	Transcription Factors interaction	Cytoplasm	Increased	JAK2/ICAM1/CCL2	JAK/STAT signaling	Brain Metastasis	[62–64]
DANCR		Cytoplasm/Nucleus	Increased	RXRA	PI3K/AKT signaling	Proliferation; Suppressor of cell differentiation	[65]
Linc01271		Nucleus	Increased	TNS1	Fibrillar adhesion; cell migration and evasion	Metastasis	[67, 68]
EGOT1		Cytoplasm	Decreased	IPTR1	pre-mRNA stability	Authophagy	[70]
MEG3	Chromatin Architect	Nucleus	Decreased	PRC2/TGFR1	TGF-β signaling pathway	Cell proliferation and progression; Autophagy	
PVT1		Cytoplasm/Nucleus	Decreased	miR-18b-5p; KLF5; c-Myc	Epigenetic Silensing; Beta Catenin signaling	Cell proliferation and progression	[77, 78]
NEAT1	Phase Separation	Nucleus	Increased	p54(NONO)	G1/S transition; Paraspeckles formation	Apoptosis and Cell cycle; Cancer stemness	[95–97]
SNHG9		Nucleus		PA/LATS1	Kinase activity	Protein aggregation	[98]
DIGIT		Nucleus		BRD3	Endoderm differentiation	Trascriptional activity	[99]

## Clinical relevance in breast cancer

### lncRNAs as a driving force of chemoresistance and metastasis

While breast cancer is treatable if detected early, the occurrence of metastatic and chemoresistant phenotypes as a result of tumor heterogeneity are a major hindrance to therapeutic intervention [101–103]. The clinical importance of lncRNAs has been demonstrated on multiple occasions through their capacity to act as promoters of tumourigenesis as well as tumor suppressors with a marked impact on disease progression and outcome through a plethora of mechanisms [104, 105]. For instance, the oncogenic properties of HOTAIR and its correlation to poor prognosis in various cancers has been well documented [49, 106, 107]. Recently researchers demonstrated that activation of HOTAIR, triggered by the uptake of signaling mediators, could promote breast cancer EMT and lung metastasis in mice via activating CDK5 signaling therefore highlighting the importance of paracrine signaling in disease progression and providing new potential therapeutic targets. Specifically, it was discovered that secretion of TGF-β1 by cancer associated fibroblasts (CAFs) led to direct binding of SMAD2-4 to the promoter of HOTAIR in breast cancer cells thus causing its expression. HOTAIR subsequently activated CDK5 signaling by recruiting the components of PRC2 complex to the promoter of CDK5RAP1 and facilitating methylation of the promoter region. HOTAIR activation was attenuated after treatment with TGF-β1 inhibitors while RNAi mediated knockdown of HOTAIR in mice abrogated the metastatic phenomenon [108].

Involved in EMT transition and metastasis, Linc-ROR has also been shown to promote estrogen-independent growth of breast cancer cells by regulating the ERK-specific DUSP7 phosphatase, thus enhancing MAPK/ERK signaling with potential implications for tamoxifen resistance [109, 110]. lncRNA TROJAN, associated with poor survival, confers CDK4/6 inhibitor resistance, and promotes proliferation in ER + breast cancer via upregulating CDK2 expression [111]. Other examples include: LINK-A which enhances AKT/HiF1-a signaling and downregulates antigen presentation gene expression, facilitating immune escape and drug resistance; AGAP2-ASI which regulates fatty acid oxidation through the formation of lncRNA/HuR/CPT1 complex to promote stemness and trastuzumab tolerance [112, 113]. Conversely, SNORD3A functions as a ceRNA to protect UMPS expression via sponging miR-185-5p, thereby sensitizing breast cancer cells to 5-FU [114]. Expression of lncRNA ANCR has been shown to reduce breast cancer cell invasion and migration capabilities by directly regulating EZH2 stability, binding to and marking it for proteasome degradation [115]. NORAD, which is under the transcriptional control of the YAP pathway, is capable of suppressing metastasis by binding to S100P and sequestering it in the cytoplasm [116].

### lncRNA implementation in precision diagnostics and therapeutics

Functional characterization and understanding of the underlying mechanisms governing lncRNA mediated regulation in human diseases could provide us with novel opportunities to revolutionize our existing arsenal of diagnostic and therapeutic tools. These transcripts possess several qualities which make them ideal for combatting cancer.

Their tissue and cell type specific expression patterns highlight their potential for use as highly accurate biomarkers [117]. LncRNAs can be utilized on their own or in complement with other biomarkers to assess a patient’s status or possible response to specific therapies and are detectable in tissue samples, such as formalin fixed paraffin embedded samples (FFPE), as well as bodily fluids [118, 119]. Isolation and studies can be achieved through classical RNA extraction protocols involving sample preparation and TRI-agents paired with RNA sequencing and qPCR [120]. While FFPE samples can be routinely obtained during biopsies, the procedure is invasive and generates heterogenous samples prone to nucleic acid degradation which can jeopardize the reliability of results [121]. A number of approaches are being developed to better detect and analyse lncRNAs in these samples including the use of target enrichment methods as well as laser micro dissection to limit the sample heterogeneity in breast cancer [121, 122]. The majority of researchers however are focusing on detecting lncRNAs or transcript fragments in bodily fluids such as serum or urine in order to discover and develop less invasive approaches, however the mechanisms which control lncRNA secretion are still poorly understood and their biological functions in cancer still under investigation [118, 120].

As mentioned before, clinical studies have revealed a link between HOTAIR and metastasis. Overexpression of this lncRNA in breast cancer samples especially of ER-positive patients has been associated with poor prognosis, indicating its potential use as a novel biomarker to predict metastasis. Additionally, serum levels of circulating HOTAIR were capable of differentiating between breast cancer patients and healthy individuals [123, 124]. Furthermore, through encapsulation and exosomal dissemination, in breast cancer lncRNAs such as ACO73352.1, HISLA, SNHG14, and SNHG16 are involved in promoting tumourigenesis, chemoresistance, as well as modulation of tumor microenvironment, piquing the interest of researchers worldwide [125–128]. Identification and detection of tumor-derived circulating exosomal lncRNAs could significantly expand our diagnostic toolkit, and provide new avenues for precision medicine. Aside from their expression patterns their sequence polymorphisms can also provide valuable insights such as those of MEG3 in breast cancer which have been associated with therapeutic efficacy, therefore making them valuable predictive markers for patients [129]. Another interesting approach would be to identify and validate metastasis associated lncRNAs, such as lnc-BM which is linked to breast cancer metastasis to the brain, which can then be used to monitor disease progression in patients.

The essential role of these transcripts in regulating malignant transformation and disease progression in breast cancer have made them extremely valuable targets in combatting this complex disease. Their overall lower levels of expression compared to protein coding genes combined with their distinguished expression patterns, make them ideal for nucleic acid-based strategies [130]. Such strategies include the use of siRNAs for targeted knockdown of transcripts via RISC as well as antisense oligonucleotides (ASOs), which hybridize with the target RNA blocking secondary structure formation and mediate degradation via RNAse-H [131]. For instance, classic RNAi has been successfully implemented for the in vivo targeting of Malat1 and HOTAIR [106]. In a similar fashion the use of MALAT1 targeting ASOs was capable of blocking breast cancer progression via MALAT1 knockdown [132–135]. Despite the efficiency of siRNAs in targeting cytoplasmic transcripts they are a bit unpredictable when it comes to nuclear lncRNAs due to problems with nuclear localization. On the other hand well designed ASOs can efficiently target lncRNAs regardless of their localization and may be better suited for dealing with aberrant nuclear lncRNAs [136]. While nucleic acid-based approaches have great potential there are still significant limitations to overcome before clinical application such as the inert instability of nucleic acids which necessitates extra molecular modifications for stability and efficiency, off target effects due to sequence pairing, immunogenicity due to immune recognition by Toll-like receptors (TLRs) [137, 138]. The main challenge however is the engineering of efficient delivery systems such as advanced nanoparticles or exosomes to ensure correct tissue and intercellular localization and avoid uptake in the wrong organs or endosome retention [138–140].

Gene editing tools such as the CRISPR-CAS9 system present many opportunities in lncRNA based therapeutics. CRISPR is being investigated both as a means by which to silence lncRNAs driving malignant transformation through CRISPRi, as well as to restore expression of transcriptionally dormant lncRNAs with tumor suppressor properties like ANCR [141, 142]. Other applications of this technique could include the fusion of CAS-9 to transcriptional repressors to target lncRNA promoters via guide RNAs for target specific transcription repression. It would be interesting to see the effects of such a system in regulating independently functional lncRNA regulatory elements like the PVT1 promoter.

However, the most promising platform for modulating oncogenic lncRNA mediated tumourigenesis would be to target their tertiary structure through which they associate with proteins, RNA, DNA and exert their regulatory effects [143]. In that context small molecular inhibitors or aptamer-based approaches could be utilized to antagonize the direct interactions between lncRNAs and their interactors [144, 145]. Indeed, a small molecular inhibitor dubbed AC1Q3QWB has been developed and tested in breast cancer derived xenografts, resulting in efficient disruption of PRC2 recruitment by HOTAIR without off target effects [146]. Another example of lncRNA structure targeting would be the TMPyP4 small molecule which disrupts the association of the NEAT1 transcript with the NONO protein through targeting of the secondary G4 structures NEAT1 uses for its interaction with NONO [28, 147]. Similar approaches are being tested for the disruption of G4 structure mediated MALAT1 interactions in associated cancers with the small molecule pyridostatin as well as peptides and aptamers [148]. The development of the RNA targeting CRISPR-CAS13 system could also be used in a similar fashion to attenuate lncRNA oncogenic functions (Table 2) [149].

**Table 2:** List of clinical relevant LncRNAs in breast cancer.

lncRNA	Mechanism/Pathway signaling	Clinical relevance	Reference
HOTAIR	EMT Transistion	Poor prognosis and metastasis	[49, 106, 107, 123, 124]
EGOT1	Induced autophagy through increased IPTR mediated autophagic signals and vesicles	Paclitaxel treatment	[70]
linc-ROR	Promote estrogen-independent growth MAPK/ERK signalin	Tamoxifen resistance	[109, 110]
Linc-TROJAN	Proliferation in in ER+ breast cancer CDK2 upregulation	CDK4/6 inhibitor resistance	[111]
LINK-A	Enhances AKT/HiF1-a signaling and downregulates antigen presentation gene expression	Immune escape and drug resistance	[112]
AGAP2-ASI	Regulates fatty acid oxidation lncRNA/HuR/CPT1 complex	Promote stemness Trastuzumab tolerance	[113]
SNORD3A	ceRNA to protect UMPS expression via sponging miR-185-5p	Sensitizing breast cancer cells to 5-FU	[114]
ANCR	Regulating EZH2 stability, binding to and marking it for proteasome degradation	Reduce breast cancer cell invasion and migration capabilities	[115]
NORAD	YAP pathway	Metastasis suppressor by binding to S100P and sequestering it in the cytoplasm	[116]
ACO73352.1, HISLA, SNHG14, and SNHG16	Modulation of tumor microenvironment	Promoting tumourigenesis, chemoresistance	[125–128]

## Concluding remarks

The advancements in the fields of transcriptomics and genomics in the last decade have elevated lncRNAs from “transcriptional noise” to functional multidimensional entities responsible for the regulation of cell fate and homeostasis. Their discovery has revealed a new complex framework of regulatory processes governing the initiation and progression of human diseases including breast cancer, with the potential to revolutionize the way we diagnose and treat it. Discovery of deregulated lncRNAs is currently ongoing, but despite the availability of cancer related genomic/transcriptomic data, very few lncRNAs have been functionally characterized due to a lack of throughput analyses of their loci and their aberrations. These transcripts have been found to function through interactions with other transcripts, DNA, and proteins facilitated by their complex tertiary structure, often assembling RNP complexes capable of forming condensates promoting LLPS [27, 150]. Mounting evidence demonstrates the importance of nuclear enriched lncRNAs in regulating chromatin organization, transcription, and DNA damage repair, with major implications for malignant transformation and metastasis in breast cancer [151].

Limitations in our understanding of lncRNA biology stem in part from the difficulty of studying their interactions in cancer. Their lack of conservation makes it difficult to establish representative mouse models, requiring the use of xenografts to bridge the gaps in our knowledge [152]. This is further complicated by the ability of some lncRNAs to perform multiple different functions within the cell, as well as having opposing roles in different cell types or disease stages [153]. lncRNA databases are being compiled to elucidate their complex roles and functions, and predictive tools for the identification of their interactions in the context of cellular type and localization as well as disease association are being developed [154–156]. The ability of lncRNAs to associate with proteins and induce the formation of phase separated droplets is a new intriguing mechanism which may broaden our understanding of the spatiotemporal control over cellular processes and provide new therapeutic avenues [150]. The utility of detecting circulating lncRNAs and the disruption of endogenous lncRNA interactions mediated by targeting of their structures through molecular inhibitors, are being investigated in the context of precision medicine. In conclusion, the discovery of these transcripts has broadened our understanding of cellular processes and provided us with potential novel biomarkers and therapeutic targets to combat breast cancer. However, throughput analyses and functional dissection of their mechanisms is still required in order to realize their true potential.
